# Bowel Wall Vascularization in Crohn’s Disease: Exploratory Comparison of Vendor-Derived Vascular Index, ImageJ-Based Quantification, and Blinded Video-Based Doppler Scoring

**DOI:** 10.3390/diagnostics16152482

**Published:** 2026-08-06

**Authors:** Paula Linhart, Wolfgang Kratzer, Mark Hänle, Jochen Klaus, Benedikt Haggenmüller

**Affiliations:** 1 Department of Internal Medicine I, University Hospital Ulm, 89081 Ulm, Germany; 2 Department of Diagnostic and Interventional Radiology, University Hospital Ulm, 89081 Ulm, Germany

**Keywords:** Crohn’s disease, intestinal ultrasound, bowel wall vascularization, superb microvascular imaging, vascular index, Doppler ultrasound, ImageJ, fecal calprotectin

## Abstract

**Background**: Assessment of inflammatory activity in Crohn’s disease (CD) remains challenging. Bowel wall vascularization is an established marker of transmural inflammation and is typically evaluated using semiquantitative ultrasound scoring systems. In contrast, quantitative vascularization analysis is not yet widely used in clinical practice. To compare these approaches, this study evaluated semiquantitative and quantitative methods in the same patient cohort. **Methods**: This prospective single-center study included 50 patients with CD and sonographically detectable bowel wall thickening. Bowel wall vascularization was assessed using power Doppler (PD), color-coded superb microvascular imaging (cSMI) and monochrome mode superb microvascular imaging (mSMI). Video sequences were independently evaluated by three blinded readers using the Limberg classification. Quantitative vascularization was assessed using a vendor-derived vascular index and retrospective ImageJ analysis. Crohn’s Disease Activity Index (CDAI) and laboratory parameters were documented. **Results**: Interobserver agreement differed between imaging modalities. Krippendorff’s α values were 0.735 (PD), 0.655 (cSMI) and 0.527 (mSMI), indicating higher reliability for PD than for the SMI techniques. According to Fleiss’ κ (0.480 (PD), 0.376 (cSMI) and 0.367 (mSMI)), agreement was moderate for PD and fair for both SMI techniques. Complete concordance among all three readers was observed in 24 (PD), 18 (cSMI), and 22 (mSMI) patients, respectively. SMI-based techniques consistently resulted in higher Limberg grades compared to PD. Exploratory analyses showed strong correlations between different quantitative vascularization parameters (ρ up to 0.898, *p* < 0.001), but only moderate correlations with fecal calprotectin (FC) (ρ up to 0.389 with *p* = 0.017). Significant negative correlations were observed between vascular indices and body mass index (BMI) and skin-to-bowel distance (ρ up to −0.614, *p* < 0.001). The same trend was observed in the semiquantitative Limberg classification. **Conclusions**: Semiquantitative assessment of bowel wall vascularization seems to be limited by interobserver variability and Doppler technique, with SMI yielding higher Limberg grades than conventional Doppler. Standardized quantitative approaches may improve objectivity, but their reproducibility requires further validation. Patient-related factors should be considered when interpreting vascularization findings.

## 1. Introduction

Assessment of inflammatory activity in Crohn’s disease remains challenging despite the availability of established clinical scores and biomarkers. Clinical indices such as the Crohn’s Disease Activity Index (CDAI) correlate only imperfectly with inflammatory burden and have limited value for assessing transmural disease activity [1,2,3]. Biomarkers such as fecal calprotectin (FC) are well established as sensitive indicators of intestinal inflammation [4,5]. However, the clinical utility of FC is limited by reduced specificity, as elevated levels may also occur in non-inflammatory bowel disease conditions and by inconsistent performance in isolated small bowel disease, where correlations with disease activity have been reported to be variable across studies [6,7,8].

Against this background, intestinal ultrasound (IUS) has gained increasing importance and is now considered a central tool for disease assessment and monitoring in Crohn’s disease [5,9,10]. Early studies from our group and others, as well as recent guidelines, identified bowel wall vascularization as a relevant sonographic marker of inflammatory activity in Crohn’s disease [2,3,11,12,13,14]. In addition to structural parameters such as bowel wall thickness, the assessment of bowel wall vascularization provides direct information on inflammatory activity and has been shown to correlate with endoscopic and histopathologic findings [2,3,11,12,14,15,16,17]. Conventional power Doppler (PD) imaging is well established for this purpose but may have limited sensitivity for low-flow vascular signals [18].

With the introduction of superb microvascular imaging (SMI), the detection of microvascular flow without contrast administration has improved substantially. Recent studies suggest that SMI-based techniques are more sensitive than conventional Doppler methods for detecting inflammation-associated vascularization and show a close relationship with endoscopic disease activity in Crohn’s disease [19,20].

In parallel, different approaches for quantifying vascularization have been developed, including system-based indices derived from color-pixel proportions and independent image-based post-processing methods such as ImageJ analysis [12,20,21,22,23,24,25]. These approaches differ substantially regarding operator dependence, image acquisition, and region of interest (ROI) definition. System-based vascular indices are derived from pixel-based quantification within a manually defined ROI and therefore remain dependent on acquisition conditions and ROI placement. Image-based methods allow standardized post-processing using predefined grayscale thresholds [22]. Previous work has shown that image analysis software-based approaches can provide reproducible quantification of vascular signals and may be comparable to other quantitative imaging methods [22,23,25]. However, in routine clinical practice, bowel wall vascularization is still commonly assessed semiquantitatively using established scoring systems such as the Limberg classification [2,3,11,15,26]. It remains unclear to what extent semiquantitative scoring, vendor-derived vascular indices, and independent image-based quantification reflect comparable or complementary aspects of bowel wall vascularization.

The reproducibility of vascularization assessment is also essential for the interpretation of Doppler-based activity markers. Previous studies have demonstrated good to excellent interobserver reliability for key intestinal ultrasound parameters in inflammatory bowel disease, particularly for disease detection, activity assessment, and bowel wall thickness [27,28,29]. Doppler-based assessment of bowel wall vascularization has also been investigated, with reported interobserver agreement ranging from fair to excellent [28,29,30]. However, comparative data on the interobserver reliability of semiquantitative vascularization assessment across different Doppler techniques remain limited.

To our knowledge, no previous study has compared vendor-derived vascularization indices, ImageJ-based quantification, and semiquantitative Limberg grading within a single Crohn’s disease cohort. In addition, the influence of technical factors and the relationship of these methods to established inflammatory markers have not been sufficiently studied.

The primary aim of the present study was to evaluate the robustness of semiquantitative bowel wall vascularization assessment using the Limberg classification in patients with Crohn’s disease. Specifically, we investigated whether the choice of Doppler technique influences the assigned Limberg score and assessed interobserver agreement of Limberg grading based on blinded video evaluation. As exploratory objectives, we compared different quantitative approaches for assessing bowel wall vascularization, including vendor-derived vascular indices and retrospective video-based quantification. Furthermore, as exploratory analyses, we investigated potential associations between both semiquantitative and quantitative vascularization parameters and patient-related technical factors such as body mass index and bowel-to-skin distance. We also explored potential correlations with clinical parameters and established laboratory markers of inflammatory activity.

## 2. Materials and Methods

### 2.1. Study Design and Ethics

This prospective single-center observational study was conducted at the University Hospital Ulm (Ulm, Germany). The study protocol was approved by the local ethics committee (No. 171/23), and all participants provided written informed consent.

### 2.2. Patient Recruitment

Between December 2023 and October 2024, consecutive patients with established Crohn’s disease presenting to the central ultrasound unit were screened for study participation. Inclusion criteria were age ≥ 18 years, confirmed diagnosis of Crohn’s disease, sonographic evidence of a single affected bowel segment, clear anatomical localization of the affected segment and bowel wall thickness ≥ 5 mm. Patients with multifocal disease, other inflammatory bowel diseases, or limited examinability were excluded. To improve comparability between imaging findings and inflammatory markers, only patients with monofocal disease were included.

### 2.3. Ultrasound Examination

All examinations were performed using an Aplio i800 ultrasound system (Canon Medical Systems, Otawara, Japan) by an experienced investigator. In 96% of cases, examinations were performed or supervised by the same investigator with extensive experience in intestinal ultrasound. A predefined examination protocol was applied. Initial assessment was performed using a convex transducer, followed by high-resolution imaging using a linear transducer. The following parameters were recorded: anatomical location, maximum bowel wall thickness, wall stratification, length of the affected segment, skin-to-bowel distance and examination conditions. To minimize bias, ultrasound examinations were performed prior to clinical assessment and CDAI calculation.

### 2.4. Assessment of Bowel Wall Vascularization

**Figure 1:**
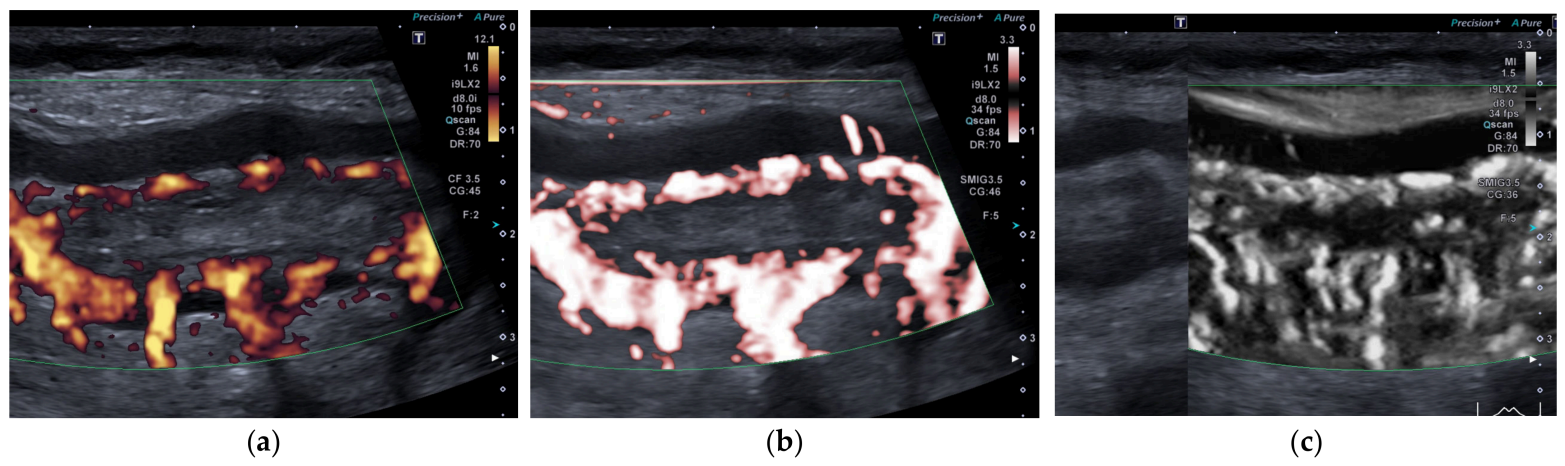
Comparison of power Doppler (**a**), color-coded SMI (**b**) and monochrome mode SMI (**c**) within the same patient; the Limberg classification of power Doppler was IV. Uncropped images are provided in the Supplementary Material.

Bowel wall vascularization was assessed using PD, color superb microvascular imaging (cSMI) and monochrome SMI (mSMI) (Figure 1). During the examination, vascularization was semi-quantitatively assessed using PD according to the Limberg classification.

### 2.5. Quantitative Vascularization Analysis

• Vendor-Derived Vascular Index (cSMI)

**Figure 2:**
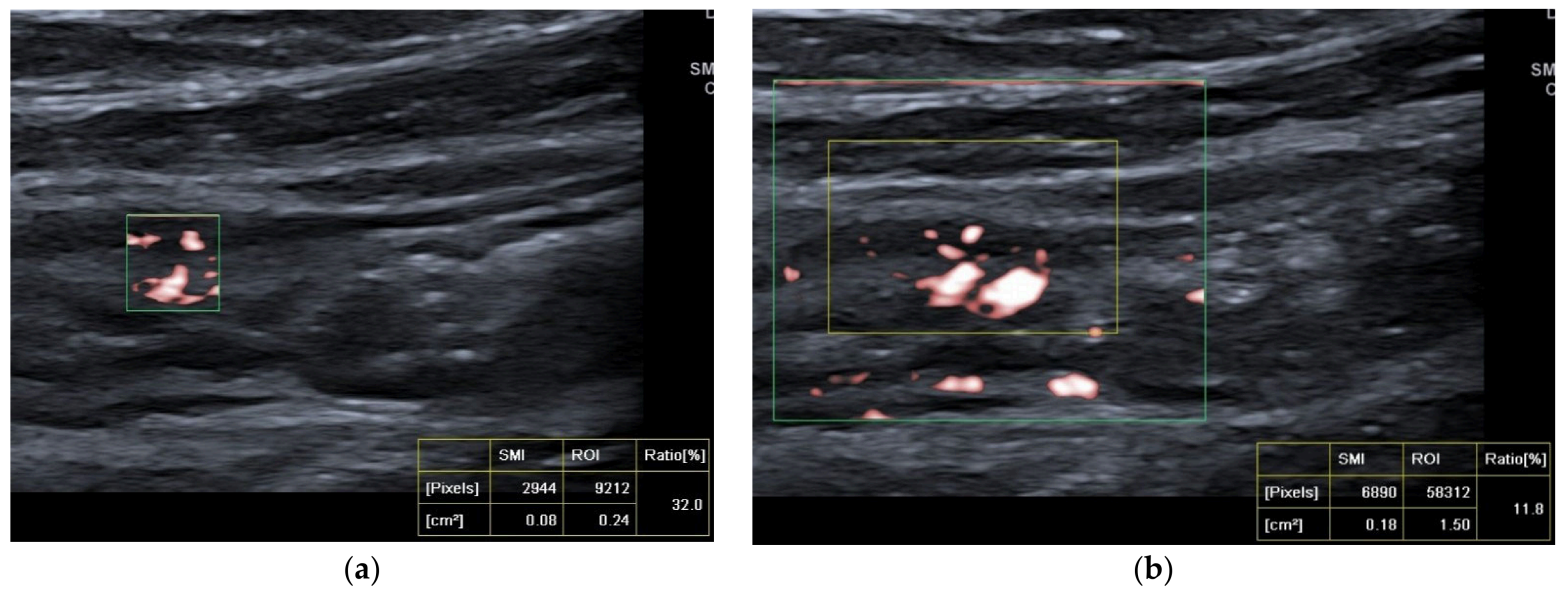
Vendor-derived vascular indices using color-coded SMI: (**a**) wall-adapted ROI; (**b**) standardized ROI of 10 × 15 mm (yellow box). The vascular index is defined as the ratio of SMI-positive pixels to total pixels. Uncropped images are provided in the Supplementary Material.

The vascular index (VI) was obtained using cSMI and calculated by the ultrasound system as the ratio of color pixels to total pixels within a manually defined ROI. Two ROI configurations were used: a bowel wall-adapted ROI (bowel wall thickness × 5 mm) and a standardized ROI (10 × 15 mm) (Figure 2). The ROI was manually placed, and pixel quantification was subsequently performed automatically by the ultrasound system. For each configuration, ten measurements per patient were performed during end-expiration or breath hold to reduce motion artifacts. The final VI was defined as the mean of these measurements. Both ROI approaches have specific advantages and limitations; therefore, both were applied to capture complementary aspects of vascularization. While the wall-adapted ROI accounts for bowel wall thickness, the standardized ROI does not include this factor but may additionally capture mesenteric vessels.

• ImageJ-Based Vascular Index (mSMI)

For exploratory quantification assessment, mSMI video sequences were analyzed retrospectively using ImageJ software (version 1.54g, NIH, Bethesda, MD, USA). Five representative frames per patient were retrospectively extracted from the recorded video sequences by a single, non-blinded investigator based on stable visualization of bowel wall vascularization, adequate visualization of the bowel wall, and minimal motion artifacts. The number of frames was selected based on the feasibility of manual analysis, which is substantially more time-consuming than the automated vendor-derived measurements. For each frame, a rectangular ROI of approximately 15 × 10 mm was placed along the bowel wall, with the size defined according to the scale displayed in the ultrasound image. Grayscale histograms (scale 0–255) were then generated. Because no externally validated grayscale threshold for mSMI-based vascularization quantification is currently available, pixels within the upper third of the grayscale range (171–255) were predefined as the grayscale component used for vascularization quantification in this exploratory approach. The VI was then calculated as the ratio of the number of pixels within this grayscale range to the total number of pixels. The final VI was defined as the mean of the five measurements. This approach represents an exploratory post-processing-based method for quantifying bowel wall vascularization and has previously been applied in a similar manner for the analysis of microvascular imaging data [22].

The two quantification approaches differed with respect to imaging modality, measurement workflow, and the number of analysed frames, which should be considered when interpreting the comparison between both indices.

### 2.6. Video-Based Assessment

Video sequences of PD, cSMI, and mSMI were recorded for each patient and anonymized. Three experienced examiners (E1–3) independently evaluated all videos using the Limberg classification. Assessments were performed in a blinded manner, in randomized order and with a minimum interval of one week between modalities. This approach was used to minimize recall bias and interobserver influence.

### 2.7. Clinical and Laboratory Parameters

Clinical data were collected using a standardized questionnaire, including demographic characteristics, disease history, and medication. Disease activity was assessed using the Crohn’s Disease Activity Index (CDAI), calculated according to Best et al. [1]. Laboratory parameters included: C-reactive protein (CRP), leukocyte count, erythrocyte sedimentation rate (ESR), hematocrit and FC (if available within 14 days of ultrasound examination).

### 2.8. Statistical Analysis

Statistical analyses were performed using SPSS (version 29.0.2, IBM Corp., Armonk, NY, USA). Descriptive statistics were calculated using medians and interquartile ranges. Associations between variables were assessed using Spearman’s rank correlation coefficient. Given the exploratory nature of these analyses, no correction for multiple testing was performed for these analyses. Bias-corrected and accelerated (BCa) 95% confidence intervals were estimated using nonparametric bootstrap resampling with 10,000 bootstrap samples. Interobserver agreement among the three readers was assessed using Fleiss’ κ with 95% confidence intervals. Since the Limberg classification represents an ordinal scale, Krippendorff’s alpha was additionally calculated to account for the magnitude of disagreement between categories. Differences in Limberg grades between power Doppler, cSMI, and mSMI were assessed for each examiner using the Friedman test for paired ordinal data. If the Friedman test was significant, post-hoc pairwise comparisons were performed with Bonferroni-adjusted *p*-values to account for multiple comparisons. A two-sided *p*-value < 0.05 was considered statistically significant. The planned sample size of 60 patients was pragmatically defined during study planning to account for potential exclusions and incomplete examinations. Given the exploratory nature and the use of multiple statistical approaches, this sample size was considered a recruitment target rather than a universal power-based estimate.

## 3. Results

### 3.1. Study Population

**Figure 3:**
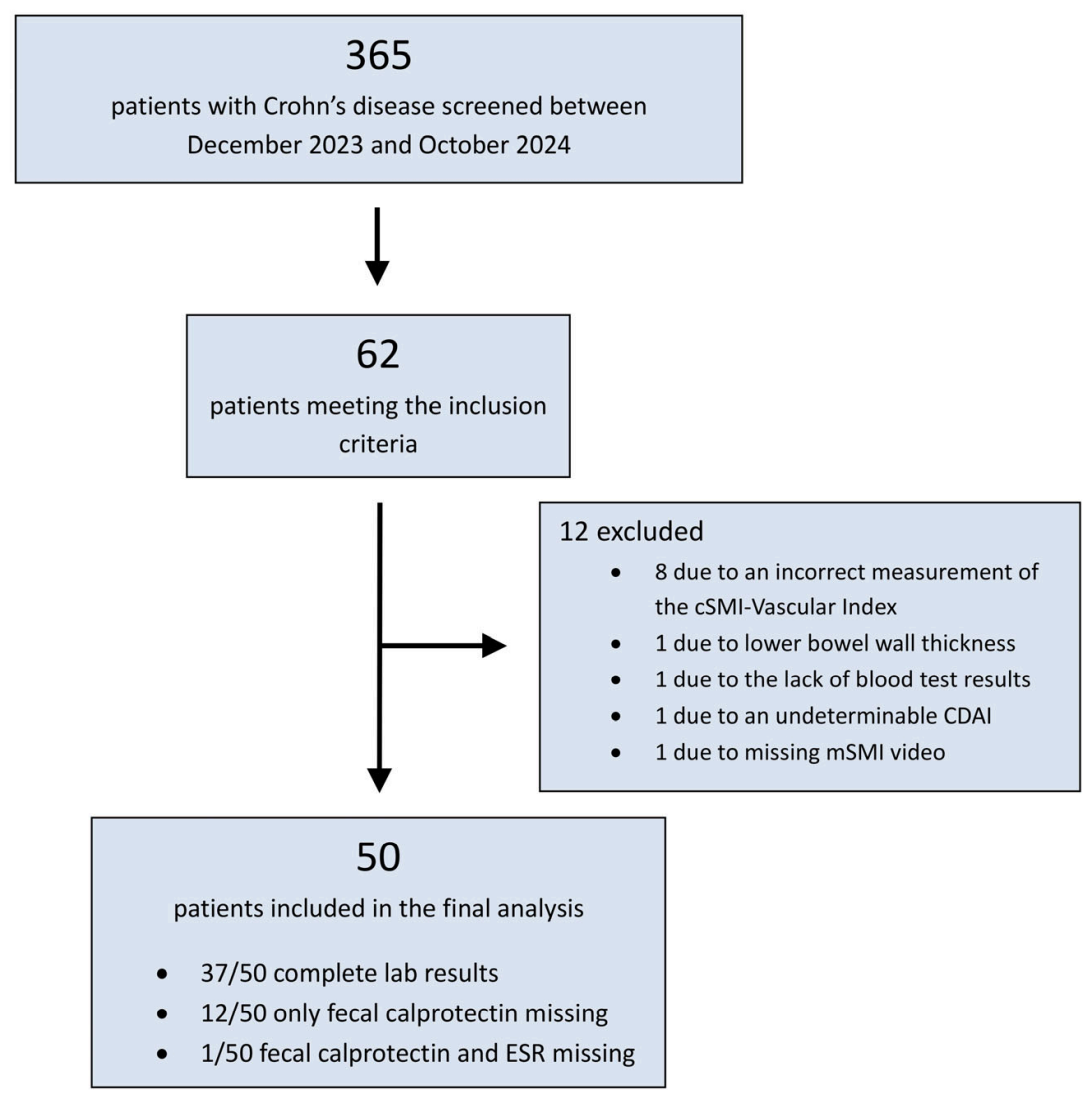
Initially screened patients and included and excluded patients.

A total of 365 patients were screened for study participation, of whom 62 patients met the inclusion criteria. The most frequent reasons for not including screened patients were the absence of bowel wall thickening, bowel wall thickening measuring less than 5 mm, or bowel wall thickening affecting multiple bowel segments. Subsequently, 12 patients were excluded from further analysis, resulting in a final cohort of 50 patients. Eight exclusions were due to incorrect cSMI-VI measurements caused by inconsistent ROI placement (Figure 3). Fecal calprotectin measurements were available in 37 patients. Patient recruitment was conducted during routine follow-up visits, and laboratory parameters included in the study were those obtained as part of standard clinical care. No additional laboratory testing was performed specifically for study purposes. In addition, during the recruitment period, temporary laboratory-related constraints resulted in intermittent unavailability of fecal calprotectin testing, leading to missing fecal calprotectin measurements in a subset of patients. In 34 patients, fecal calprotectin was measured on the day of the ultrasound examination, in the remaining three patients, the interval between measurement and ultrasound was 6, 7, and 8 days, respectively. Baseline characteristics of the study population are summarized in Table 1.

### 3.2. Interobserver Agreement of Video-Based Assessment

Interobserver agreement for the semiquantitative assessment of bowel wall vascularization using the Limberg classification was overall fair to moderate across all imaging modalities. Based on Fleiss’ κ, agreement was highest for power Doppler (κ = 0.480, 95% CI 0.379–0.581), with all three examiners assigning the same Limberg stage in 24 patients (48%). Both SMI-based techniques showed slightly lower unweighted agreement (cSMI: κ = 0.376, 95% CI 0.275–0.477; mSMI: κ = 0.367, 95% CI 0.248–0.487), with complete agreement among all three examiners in 18 patients (36%) for cSMI and 22 patients (44%) for mSMI, respectively. Krippendorff’s alpha was additionally calculated to account for the magnitude of disagreement between categories, resulting in α values of 0.735 for power Doppler, 0.655 for cSMI, and 0.527 for mSMI. According to commonly used interpretation criteria, none of the modalities reached the threshold of α ≥ 0.80 proposed for strong reliability, although power Doppler showed the highest reproducibility [32]. A descriptive analysis of individual reader assessments revealed no systematic deviation of any single reader toward higher or lower Limberg stages across the evaluated imaging modalities. Overall, these findings indicate moderate reproducibility of video-based vascularization assessment, with a tendency toward lower interobserver agreement for SMI-based techniques compared with conventional power Doppler imaging (Table 2).

### 3.3. Comparison of Vascularization Grading Across Doppler Modalities

**Figure 4:**
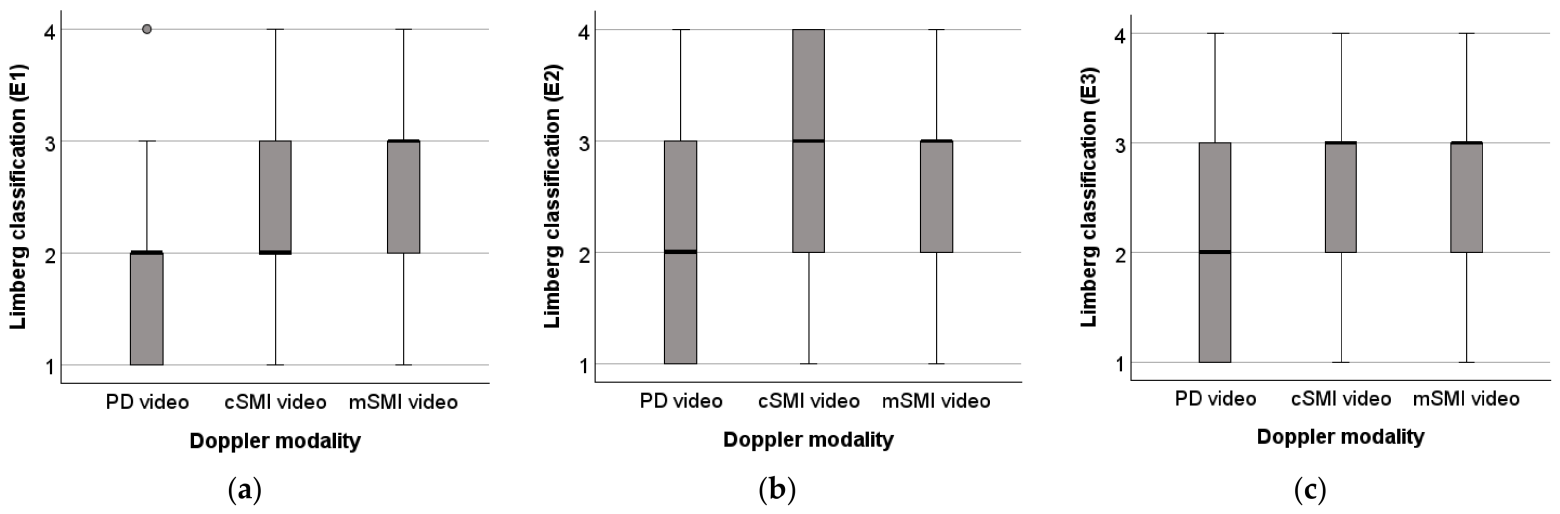
Boxplots of the Limberg classification in different Doppler modalities: (**a**) classification by examiner 1 (E1); (**b**) classification by examiner 2 (E2); (**c**) classification by examiner 3 (E3); *n* = 50 in each modality. Individual points beyond the whiskers indicate values outside the 1.5 × IQR range.

A Friedman test demonstrated significant differences in Limberg grades among power Doppler, cSMI, and mSMI for all three examiners (E1: χ^2^(2) = 46.49, *p* < 0.001; E2: χ^2^(2) = 32.99, *p* < 0.001; E3: χ^2^(2) = 10.89, *p* = 0.004). Mean ranks increased from power Doppler to cSMI and mSMI for all three examiners, indicating a shift toward higher Limberg grades with SMI-based imaging. Bonferroni-adjusted post-hoc pairwise comparisons showed significantly higher Limberg grades for both cSMI and mSMI compared with power Doppler for examiners 1 (cSMI vs. PD: *p* = 0.008; mSMI vs. PD: *p* < 0.001) and 2 (cSMI vs. PD: *p* < 0.001; mSMI vs. PD: *p* = 0.001). For E3, only mSMI yielded significantly higher Limberg grades than power Doppler (*p* = 0.037), whereas the difference between cSMI and power Doppler was not significant (*p* = 0.363). No significant differences were observed between cSMI and mSMI for any examiner. This shift towards higher vascularization grades is illustrated in Figure 4.

### 3.4. Correlation Between Quantitative Vascularization Parameters

**Figure 5:**
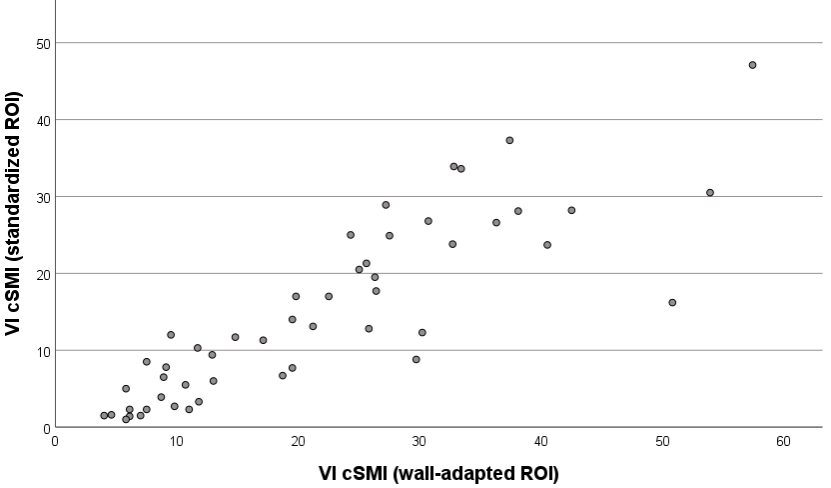
Relationship between VI cSMI (%) using a wall-adapted ROI and VI cSMI (%) using a standardized ROI. Individual measurements are shown (*n* = 50). A positive association between the two variables is observed.

**Figure 6:**
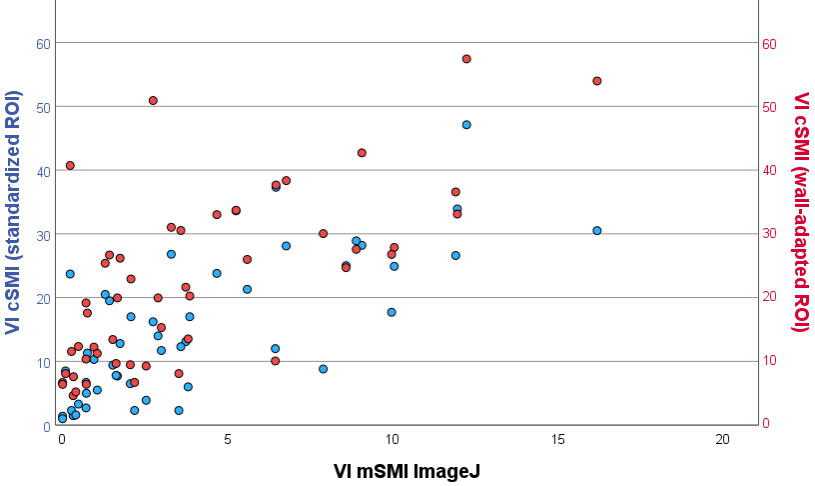
Relationship between the two vendor-derived vascular indices (VI cSMI (%) wall-adapted ROI and VI cSMI (%) standardized ROI) and the monochrome SMI VI (%) derived using ImageJ. Individual measurements are shown (*n* = 50). Data points are color-coded according to the ROI type (blue: standardized ROI; red: wall-adapted ROI).

Quantitative vascularization parameters showed strong correlations with each other. The strongest correlation was observed between the two vendor-derived cSMI vascular indices (standardized ROI 10 × 15 mm and bowel wall-adapted ROI; Spearman ρ = 0.898, *p* < 0.001; Figure 5). The vendor-derived cSMI vascular index (standardized ROI 10 × 15 mm) also demonstrated a strong correlation with the ImageJ-based mSMI vascular index (ρ = 0.753, *p* < 0.001; Figure 6). Similarly, the bowel wall-adapted cSMI ROI showed substantial correlation with the ImageJ-based measurements (ρ = 0.688, *p* < 0.001; Figure 6), indicating good agreement between different quantitative approaches (Table 3).

### 3.5. Association Between Quantitative Vascularization Parameters and Limberg Classification

**Figure 7:**
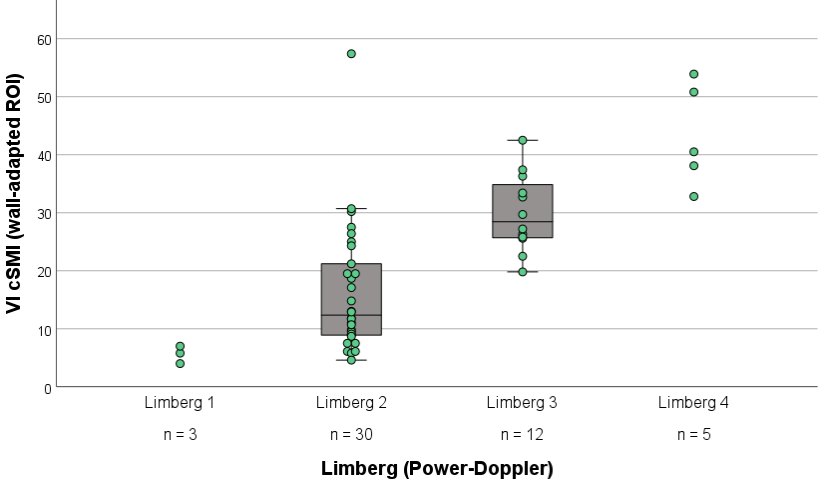
Boxplot showing the VI cSMI (%) using a wall-adapted ROI depending on the Limberg classification. The individual measurements are presented (Limberg 1: *n* = 3; Limberg 2: *n* = 30; Limberg 3: *n* = 12; Limberg 4: *n* = 5).

**Figure 8:**
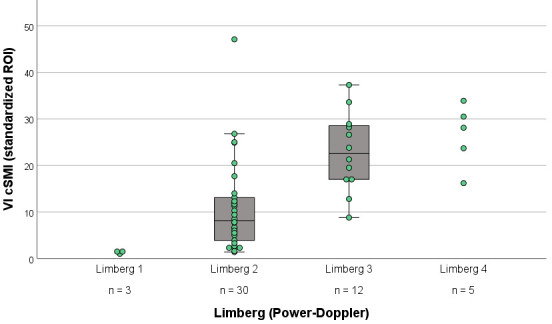
Boxplot showing the VI cSMI (%) using a standardized ROI depending on the Limberg classification. The individual measurements are presented (Limberg 1: *n* = 3; Limberg 2: *n* = 30; Limberg 3: *n* = 12; Limberg 4: *n* = 5).

**Figure 9:**
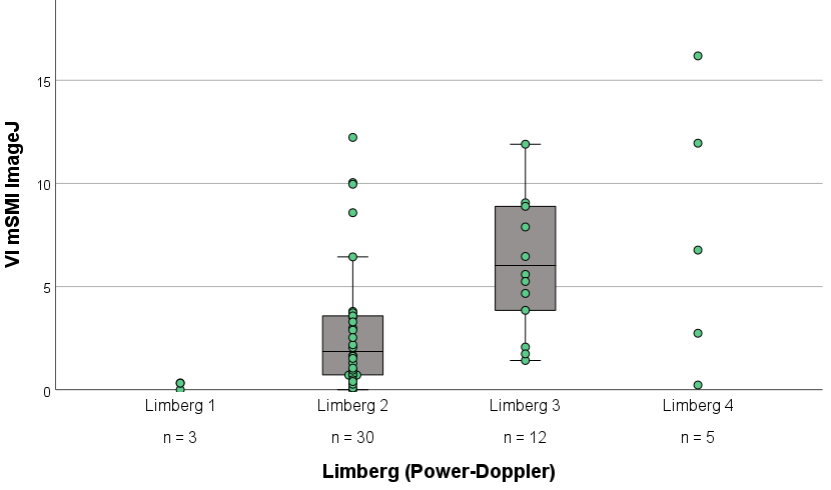
Boxplot showing the ImageJ-based mSMI vascular index (%) grouped by the Limberg classification. The individual measurements are presented (Limberg 1: *n* = 3; Limberg 2: *n* = 30; Limberg 3: *n* = 12; Limberg 4: *n* = 5).

The quantitative vascularization parameters were also compared with the Limberg scores determined using power Doppler during the examination, which served as the primary reference, as power Doppler Limberg grading is an established clinical assessment and was prospectively acquired during the live examination. Higher vascularization values were associated with increasing Limberg scores in all quantitative modalities (Figure 7, Figure 8 and Figure 9). As the Limberg subgroups were unbalanced (grade 1: *n* = 3; grade 4: *n* = 5), individual data points and the sample size for each subgroup are presented in the figures to illustrate the underlying data distribution.

Exploratory analyses using the retrospectively assigned video-based cSMI and mSMI Limberg grades also showed this tendency towards increasing vascular indices with increasing Limberg grade.

### 3.6. Association with Inflammatory Markers

**Figure 10:**
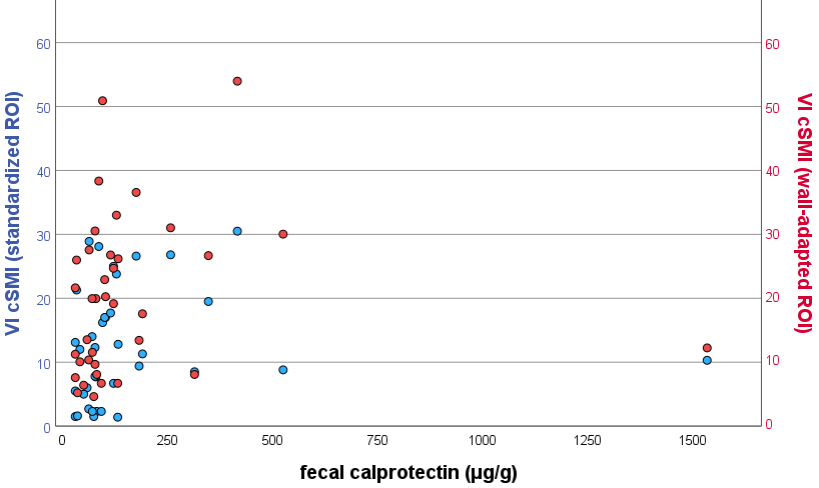
Relationship between the two vendor-derived vascular indices (VI cSMI (%) wall-adapted ROI and VI cSMI (%) standardized ROI) and the fecal calprotectin. Individual measurements are shown (*n* = 37). Data points are color-coded according to the ROI type (blue: standardized ROI; red: wall-adapted ROI).

Only weak to moderate correlations were observed between vascularization parameters and fecal calprotectin. Both vendor-derived vascular indices (wall-adapted ROI and standardized ROI) showed a statistically significant but moderate correlation with FC (standardized ROI: ρ = 0.346, 95% CI [0.056, 0.598], *p* = 0.036; wall-adapted ROI: ρ = 0.389, 95% CI [0.096, 0.617], *p* = 0.017; Figure 10). However, the ImageJ-based vascular index did not show a significant association (ρ = 0.100, 95% CI [−0.251, 0.434], *p* = 0.555). No significant correlation was observed between vascularization parameters and CDAI. As no correction for multiple testing was applied, these findings should be interpreted as exploratory.

### 3.7. Influence of Patient-Related Factors

**Figure 11:**
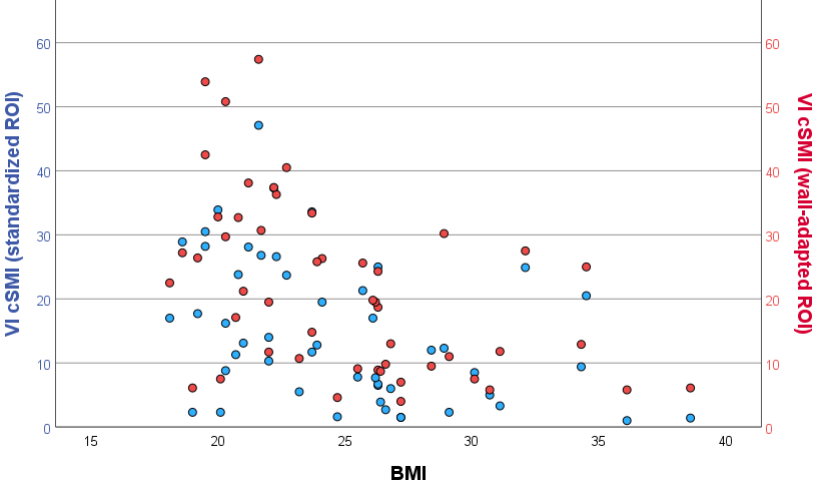
Relationship between the two vendor-derived vascular indices (VI cSMI (%) wall-adapted ROI and VI cSMI (%) standardized ROI) and the body mass index (BMI) in kg/m^2^. The individual measurements are presented (*n* = 50). Data points are color-coded according to the ROI type (blue: standardized ROI; red: wall-adapted ROI).

**Figure 12:**
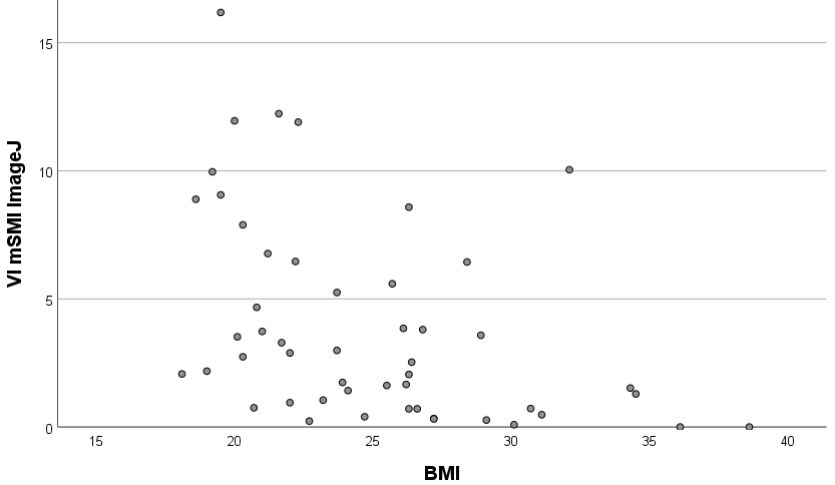
Relationship between BMI (kg/m^2^) and the ImageJ-based mSMI vascular index (%). Individual measurements are shown (*n* = 50).

**Figure 13:**
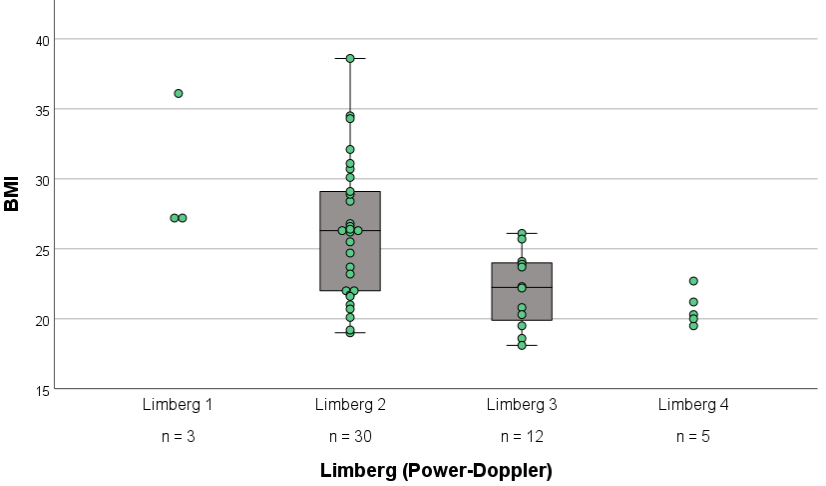
Boxplot showing the BMI (kg/m^2^) grouped by the Limberg classification. The individual measurements are presented (Limberg 1: *n* = 3; Limberg 2: *n* = 30; Limberg 3: *n* = 12; Limberg 4: *n* = 5).

The study cohort covered a broad BMI range (18.1–38.6 kg/m^2^; median 24.0 kg/m^2^), with 23 of 50 patients (46%) having a BMI > 25 kg/m^2^, including 8 patients (16%) with obesity (BMI > 30 kg/m^2^). Quantitative vascularization measurements were significantly associated with technical and patient-related factors. The vendor-derived vascular index showed a strong negative correlation with body mass index (wall-adapted ROI: ρ = −0.510, 95% CI [−0.710, −0.256], *p* < 0.001, standardized ROI: ρ = −0.489, 95% CI [−0.704, −0.220], *p* < 0.001; Figure 11) and a moderate negative correlation with skin-to-bowel distance (wall-adapted ROI: ρ = −0.396, 95% CI [−0.631, −0.100], *p* = 0.004, standardized ROI: ρ = −0.355, 95% CI [−0.602, −0.058], *p* = 0.011). Similar associations were seen considering the mSMI VI (Figure 12) where ImageJ was used (BMI: ρ = −0.535, 95% CI [−0.741, −0.256], *p* < 0.001; skin-to-bowel distance: ρ = −0.614, 95% CI [−0.785, −0.376], *p* < 0.001). Moreover, a similar trend was also observed in the Limberg scores (Figure 13).

## 4. Discussion

The present study demonstrates that semiquantitative assessment of bowel wall vascularization using the Limberg classification shows variable interobserver agreement in blinded video-based evaluation. Conventional power Doppler showed the highest agreement between observers, whereas both SMI-based techniques demonstrated lower interobserver reliability, with fair to moderate agreement according to Fleiss’ κ values. According to Krippendorff’s α, only PD reached the threshold for tentative conclusions, whereas both SMI techniques remained below this level [32]. Previous studies have also investigated the interobserver reliability of bowel wall vascularization assessment in inflammatory bowel disease. Fraquelli et al. reported interobserver agreement ranging from fair to excellent for power Doppler-based assessment of bowel wall vascularization in Crohn’s disease, based on a dichotomous assessment of the presence or absence of bowel wall flow signals and pairwise comparisons between six observers [29]. De Voogd et al. demonstrated substantial agreement across six observers using a four-category modified Limberg score assessed with color Doppler in patients with ulcerative colitis (UC) [28]. Direct comparison with the present results is, however, limited by methodological differences, including the use of different Doppler techniques, grading systems, agreement statistics, and study designs. In addition, our study evaluated the interobserver agreement of semiquantitative Limberg grading across different Doppler techniques using a blinded video-based assessment. A similar approach was applied by Haberkamp et al. in patients with UC, who investigated interobserver agreement of a semiquantitative SMI score using image and video material. High interobserver agreement was observed for both color Doppler imaging and SMI [30].

Although the Limberg classification provides a simple and clinically intuitive framework for describing bowel wall vascularization, the categorization into discrete grades may contribute to uncertainty, particularly at intermediate vascularization levels where differences between adjacent categories may be subtle. This interpretation is further supported by the findings of de Voogd et al., who demonstrated increased interobserver agreement when adjacent Limberg categories were merged, suggesting that the finer stratification of vascularization into multiple categories may reduce interobserver reliability [28]. This may explain why complete agreement between all three readers was achieved in less than half of cases, even with the modality showing the highest interobserver consistency. These findings indicate a susceptibility of Limberg grading to operator dependency, which should be taken into account when interpreting and comparing results, particularly when applied across different Doppler imaging techniques.

A key finding of our study was the consistent shift towards higher Limberg grades when using SMI compared with conventional power Doppler. Both cSMI and mSMI resulted in significantly higher vascularization scores for most examiners, suggesting increased sensitivity of SMI for detecting low-flow microvascular signals. This observation agrees with previous studies demonstrating improved visualization of microvascular flow using SMI compared to conventional Doppler techniques [19,30]. Haberkamp et al. demonstrated that SMI was superior to conventional Doppler imaging for identifying endoscopic remission in patients with UC, achieving excellent diagnostic accuracy and improved discrimination between endoscopic remission and low-grade disease activity [30]. Recent studies have also shown that SMI-derived parameters are useful to detect endoscopic active disease [20]. The observed shift towards higher Limberg grades is likely related to the technical characteristics of SMI, which is designed to suppress motion-related artifacts and enhance visualization of low-flow signals and small vessels within the bowel wall. These technical differences may influence the visualization of vascular signals compared with conventional Doppler techniques and thereby affect the assignment of Limberg grades and grading comparability. The detection of additional low-flow signals may also contribute to greater variability in how vascular findings are categorized by different observers, potentially explaining the slightly lower interobserver agreement observed for SMI-based techniques in our study. However, this potential effect was not observed in the study by Haberkamp et al., who reported high interobserver agreement for SMI-based vascularization assessment in patients with UC [30].

These findings also have important implications for the clinical interpretation of vascularization grading. Our results suggest that Limberg grades obtained with different Doppler modalities should not be used interchangeably or directly compared. In particular, an apparent increase in Limberg grade during follow-up may be caused using another Doppler modality rather than representing a true change in disease activity. Therefore, longitudinal assessment of disease activity requires consistent application of the same imaging technique and standardized acquisition protocols.

This study took an exploratory approach by evaluating different quantitative methods, including vendor-derived vascular indices and retrospective ImageJ-based quantification. Quantitative vascularization parameters showed strong positive correlations across all evaluated methods, suggesting that the evaluated methods capture related aspects of bowel wall vascularization. The strongest correlation was observed between the two vendor-derived cSMI vascular indices (Spearman’s ρ = 0.898, *p* < 0.001). Correlations between the vendor-derived indices and the retrospective ImageJ-based measurements were also strong (ρ = 0.688–0.753, *p* < 0.001), although slightly lower. The lower correlations observed between the vendor-derived and ImageJ-based measurements may reflect the methodological differences between the approaches, including the use of mSMI versus cSMI, ROI definition, the number of measurements, frame selection and image processing.

Furthermore, all quantitative vascularization parameters increased with higher Limberg scores, supporting the association between quantitative vascularization measures and the established semiquantitative assessment, although this finding should be interpreted with caution because of the small and unequal subgroup sizes. Whether quantitative vascularization assessment provides improved interobserver reliability and greater objectivity compared with established semiquantitative methods remains to be determined and warrants further investigation. A study by Lee et al. already demonstrated excellent interobserver reliability for the SMI-derived Vascularity Index in the evaluation of breast masses, supporting the reproducibility of SMI-VI as a quantitative parameter for sonographic vascularity assessment [25]. However, operator-related factors may also play a role in quantitative measurements, particularly regarding ROI placement. In our cohort, inconsistent ROI positioning resulted in incorrect VI measurements and the exclusion of 8 patients.

The role of bowel wall vascularization as a marker of inflammatory activity in Crohn’s disease has been recognized for more than two decades. Early studies demonstrated that Doppler-detected vascularization correlates with histological inflammation and disease activity, whereas clinical indices such as CDAI show limited correlation [2,3]. Our findings confirm and extend these observations. In line with previous reports, no significant association was observed between vascularization parameters and CDAI, supporting the concept that clinical indices insufficiently reflect transmural inflammation [2,3]. Nevertheless, as our study population predominantly consisted of patients with clinically inactive disease, the applicability of these findings to patients with more active or extensive Crohn’s disease requires further investigation. Furthermore, in the absence of longitudinal follow-up and an independent endoscopic or histopathological reference standard, it remains unclear whether increased Doppler-detected vascularization in clinically inactive patients reflects subclinical inflammatory activity with potential prognostic significance or vascular phenomena unrelated to active inflammation.

The increasing importance of intestinal ultrasound in disease monitoring has been emphasized in recent guidelines, which highlight vascularization as a key parameter alongside bowel wall thickness [13]. Furthermore, recent studies have demonstrated that ultrasound parameters correlate with histopathological findings and may predict disease activity [3,16,17]. In this context, the relationship between quantitative vascularization assessment and biochemical markers of inflammation is of particular interest. In our study, only the vendor-derived vascular indices of cSMI demonstrated statistically significant correlations with fecal calprotectin (standardized ROI: ρ = 0.346, *p* = 0.036; wall-adapted ROI: ρ = 0.389, *p* = 0.017), whereas the correlation for the ImageJ-based approach using mSMI did not reach statistical significance. These findings align with previous observations suggesting that fecal calprotectin may complement imaging-based parameters to improve the assessment of disease activity [4]. While FC primarily reflects mucosal inflammation, ultrasound-based vascularization assessment provides information on transmural inflammatory processes [8,10]. However, the correlations observed in our study were based on a subset of 37 patients and should be interpreted with caution, as they may not remain statistically significant after correction for multiple testing. They should therefore be considered hypothesis-generating and require confirmation in larger prospective studies.

A particularly relevant finding of our study is the association between patient-related factors and bowel wall vascularization assessment. Both vendor-derived and ImageJ-based vascularization parameters showed significant negative correlations with body mass index and skin-to-bowel distance. A similar trend was also observed for the semiquantitative Limberg grades, indicating that these associations are not restricted to quantitative approaches but may represent a broader consideration in ultrasound-vascularization assessment of patients with CD. Multiple patient-related, technical, and disease-specific factors should be considered when interpreting this association and vascularization measurements. A possible explanation for this finding is that greater tissue depth may reduce Doppler signal detectability, resulting in lower measured vascularization signals in patients with higher BMI. Previous studies have demonstrated that increasing body mass index is associated with reduced ultrasound image quality due to greater tissue attenuation, decreased signal penetration, and impaired visualization of vascular structures [34,35]. Therefore, the observed associations may partly reflect technical limitations affecting Doppler signal acquisition. However, given the close relationship between BMI and skin-to-bowel distance, as well as potential associations with disease location, previous surgery, bowel wall thickness, and inflammatory activity, these findings cannot establish an independent technical effect. Furthermore, lower BMI may also be related to more active disease through mechanisms such as malnutrition and weight loss [36,37]. Thus, BMI and skin-to-bowel distance should be considered potential confounding factors when interpreting Doppler-based vascularization parameters. These factors may contribute to lower detected vascularization signals; however, without an independent reference standard, the extent to which this represents true underestimation of vascular activity and inflammatory burden remains uncertain. The present findings should therefore be regarded as exploratory associations requiring confirmation in future studies with adjusted analyses.

### Strengths and Limitations

This study has several strengths. It provides an exploratory comparison of multiple vascularization assessment methods within the same patient population. Only patients with a single affected bowel segment were included, which improves comparability with the inflammatory parameters. The use of blinded video-based assessment by independent readers strengthens the validity of our findings.

However, several limitations should be acknowledged. First, the sample size is relatively small, particularly for the subgroup with available fecal calprotectin data. Especially the relatively strict inclusion criteria, particularly restriction to monofocal disease, contributed to the limited sample size but improved comparability between imaging findings and inflammatory markers. Fecal calprotectin measurements were not available in all patients because stool sampling was performed as part of routine clinical care rather than mandated by the study protocol. Moreover, in three patients, fecal calprotectin was not measured on the same day as the ultrasound examination. Therefore, we cannot exclude the possibility that treatment modifications occurred between the two assessments, potentially affecting the comparability of the results. In addition, the study population largely consisted of patients with clinically inactive disease, which may limit the generalizability of our findings to patients with more active or extensive Crohn’s disease. The heterogeneity of disease locations and previous surgical status represents a limitation, particularly for analyses evaluating associations between quantitative vascularization parameters and clinical, laboratory, and patient-related factors, as anatomical and postsurgical differences may influence vascularization measurements. Furthermore, no systematic endoscopic or histopathological reference standard was available. Therefore, this study cannot determine whether higher SMI-based grades reflect increased sensitivity for true inflammatory activity or rather the detection of additional low-flow vascular signals. Consequently, diagnostic accuracy and clinical superiority of one Doppler technique over another cannot be established. A limitation of the quantitative comparison is that vendor-derived and ImageJ-based measurements were obtained using different SMI acquisition modes and analysis workflows. Therefore, differences between approaches may also be related to acquisition parameters, ROI definition, or image processing rather than the quantification method or Doppler modality alone. Moreover, as the grayscale threshold and image selection criteria were not externally validated and reproducibility analyses for frame selection, ROI placement, and vascularization index calculation were not performed, the ImageJ-based grayscale analysis should be considered an exploratory approach requiring further validation. In addition, frame selection was performed retrospectively by a single, non-blinded investigator, which may have introduced observer-related bias. Furthermore, two of the 50 ultrasound examinations (4%) were performed by experienced investigators other than the primary examiner because the study was conducted within routine clinical practice. Although the proportion was small, this may have introduced minor examiner-related variability. Finally, quantitative measurements may be influenced by technical and examiner-dependent factors, which may limit their generalizability.

## 5. Conclusions

In conclusion, semiquantitative assessment of bowel wall vascularization using the Limberg classification seems to be limited by interobserver variability and dependence on the applied Doppler technique. SMI-based imaging results in higher Limberg grades, limiting direct comparability between modalities. Whether quantitative approaches can improve standardization and facilitate more objective assessment of vascularity requires further investigation. Both semiquantitative and quantitative vascularization assessments may be affected by patient-related factors, which should be considered during interpretation. A combined use of semiquantitative and quantitative approaches, together with established biomarkers, may improve the assessment of inflammatory activity in clinical practice.

## Figures and Tables

**Table 1:** Patients’ characteristics.

Parameter	*n* = 50 *
Age (years), median (IQR)	41 (31.5–56)
Sex, *n* (%)	
Men	24 (48%)
Women	26 (52%)
BMI (kg/m^2^), median (IQR)	24 (21–27.2)
Disease duration (years), median (IQR)	11 (5.5–20), *n* = 49 *
Previous surgery, *n* (%)	24 (48%)
Disease location, *n* (%)	
Terminal ileum	20 (40%)
Neoterminal ileum	20 (40%)
Other small bowel locations, *n* (%)	2 (4%)
Sigma	3 (6%)
Ascending colon	2 (4%)
Descending colon	2 (4%)
Transverse colon	1 (2%)
Bowel wall thickness (mm), median (IQR)	6.0 (5.8–7.0)
CRP (mg/L), median (IQR)	3.5 (1.2–7.4)
Fecal calprotectin (µg/g), median (IQR)	92 (62.5–153.5), *n* = 37 *
CDAI, median (IQR)	77 (33–104)
Inactive disease ^1^ (CDAI < 150), *n* (%)	45 (90%)
Mild disease ^1^ (CDAI 150–220), *n* (%)	5 (10%)

^1^ CDAI categorization: <150 inactive diseases; 150–220 mild disease; 220–450 moderate disease; >450 severe disease [31]. * Different sample sizes (*n*) for disease duration and fecal calprotectin.

**Table 2:** Interobserver agreement.

Modality	Fleiss’ Kappa	Interpretation ^1^	Krippendorff’s Alpha
power Doppler	0.480, 95% CI [0.379, 0.581]	Moderate agreement	0.735, 95% CI [0.667, 0.799]
cSMI	0.376, 95% CI [0.275, 0.477]	Fair agreement	0.655, 95% CI [0.565, 0.738]
mSMI	0.367, 95% CI [0.248, 0.487]	Fair agreement	0.527, 95% CI [0.406, 0.639]

^1^ Interpretation of Fleiss’ Kappa from Landis and Koch (1977) [33].

**Table 3:** Correlation analysis of vascular indices.

Parameter Pair		Spearman ρ, 95% CI	*p*-Value
cSMI VI (standardized ROI)	cSMI VI (wall-adapted ROI)	0.898, [0.838, 0.929]	<0.001
mSMI VI (ImageJ)	cSMI VI (standardized ROI)	0.753, [0.579, 0.866]	<0.001
mSMI VI (ImageJ)	cSMI VI (wall-adapted ROI)	0.688, [0.476, 0.832]	<0.001

## Data Availability

Data supporting the findings of this study are available from the corresponding author upon reasonable request. The data are not publicly available due to privacy and ethical restrictions involving patient information.

## Supplementary Materials

The following supporting information can be downloaded at: https://www.mdpi.com/article/10.3390/diagnostics16152482/s1.

## Abbreviations

The following abbreviations are used in this manuscript:

CD	Crohn’s disease
CDAI	Crohn’s Disease Activity Index
CRP	C-reactive protein
cSMI	color-coded superb microvascular imaging
ESR	erythrocyte sedimentation rate
E1	Examiner 1
E2	Examiner 2
E3	Examiner 3
FC	Fecal calprotectin
IUS	intestinal ultrasound
mSMI	monochrome mode superb microvascular imaging
PD	power Doppler
ROI	Region of interest
SMI	Superb microvascular imaging
UC	Ulcerative Colitis
VI	Vascular index
